# Non-periodic oscillatory deformation of an actomyosin microdroplet encapsulated within a lipid interface

**DOI:** 10.1038/srep18964

**Published:** 2016-01-12

**Authors:** Yukinori Nishigami, Hiroaki Ito, Seiji Sonobe, Masatoshi Ichikawa

**Affiliations:** 1 Department of Physics, Graduate School of Science, Kyoto University, Kyoto 606-8502, Japan; 2 Department of Life Science, Graduate School of Life Science, University of Hyogo, Harima Science Park City, Hyogo 678-1297, Japan

## Abstract

Active force generation in living organisms, which is mainly involved in actin cytoskeleton and myosin molecular motors, plays a crucial role in various biological processes. Although the contractile properties of actomyosin have been extensively investigated, their dynamic contribution to a deformable membrane remains unclear because of the cellular complexities and the difficulties associated with *in vitro* reconstitution. Here, by overcoming these experimental difficulties, we demonstrate the dynamic deformation of a reconstituted lipid interface coupled with self-organized structure of contractile actomyosin. Therein, the lipid interface repeatedly oscillates without any remarkable periods. The oscillatory deformation of the interface is caused by the aster-like three-dimensional hierarchical structure of actomyosin inside the droplet, which is revealed that the oscillation occurs stochastically as a Poisson process.

Eukaryotic cells show a variety of shapes to carry out the highly specific biological functions^1^,^2^,^3^. In addition, their shapes are not static but successively changing in response to internal and external environmental factors in ordinary biological phenomena^4^,^5^,^6^. As is well known, actomyosin (actin and myosin) proteins play critical roles in these deformations^5^,^7^,^8^,^9^,^10^. Myosin II protein, a motor protein which runs directionally on an actin filament, is involved in the force generation in cellular morphological changes. In a physiological condition, myosin II molecules assemble into bipolar thick filament. Since each end of the bipolar thick filament runs toward the opposite direction in a cooperative manner, coexistence of actin filaments and myosin thick filaments leads contraction of the network of the actomyosin complex^11^,^12^. The rheological and non-equilibrium property of actomyosin network during contraction have been measured by passive- and active-microrheology methods^13^,^14^,^15^. This contractile force generated by the actomyosin must be transmitted to a cellular membrane to exhibit deformation of the cell shape and migration of the cell body. Although various types of regulation factors concern with the actomyosin, lots of active motion of cell basically originates in the force generation from actomyosin, such as a cleavage furrow induced by contractile ring during cytokinesis^7^,^8^,^11^, membrane protrusion caused by contraction of cell cortex during bleb-driven locomotion^9^,^10^, and retraction at posterior region of cell during cell locomotion^16^,^17^,^18^. On the other hand, to elucidate the native properties of the actomyosin as a soft material, *in vitro* systems with only a small number of cellular components have been developed. For example, hierarchical structure of actin filaments were presented in two-dimensional motility assay systems, where the actin filaments are driven by myosin motors fixed on a solid substrate^19^,^20^. In the case of initially dispersed actin and myosin in a solution, the actin filaments and myosin thick filaments form the actomyosin complex, and further aggregate into an aster-like hierarchical structure in bulk^21^ and inside a cell-sized lipid vesicle^22^. As a more realistic model system, Carvalho *et al.* reported that cortex-like structure of actomyosin that is anchored to the inner surface or outer surface of lipid vesicles by biotin-avidin interaction, results in peeling of the actomyosin cortex from the inner or outer surfaces of the vesicles, crushing of the vesicle (outside-geometry), or spherical aggregation inside the vesicle (inside-geometry)^23^.

These experiments have verified the active and contractile properties of the system composed of actin and myosin, and their characteristic structural formation under certain boundary conditions. However, the dynamic deformation of membranes caused by the continuous remodeling of the contractile actomyosin, where the actomyosin distribution and contractility should interact with the membrane elasticity and interfacial tension, remains as a crucial open question. It is because of a technical problem: Suitable connection between a lipid membrane and contractile actomyosin was difficult to reconstitute *in vitro*. In addition, the difficulty in encapsulation of actin and myosin at biologically-relevant concentrations of the order of mg/ml inside a lipid bilayer vesicles have been one of the major obstacles to reconstitute the connection^24^,^25^. In the present study, we have successfully reconstituted a simple model system with a cell-sized deformable lipid interface that is connected to contractile actomyosin in a non-specific manner. We have demonstrated, for the first time, the dynamical repetitive deformation of the spherical interface, which continued for several tens of minutes, coupled with the structural formation of the encapsulated actin and myosin.

## Results

### Reconstitution of an actomyosin droplet

**Figure 1:**
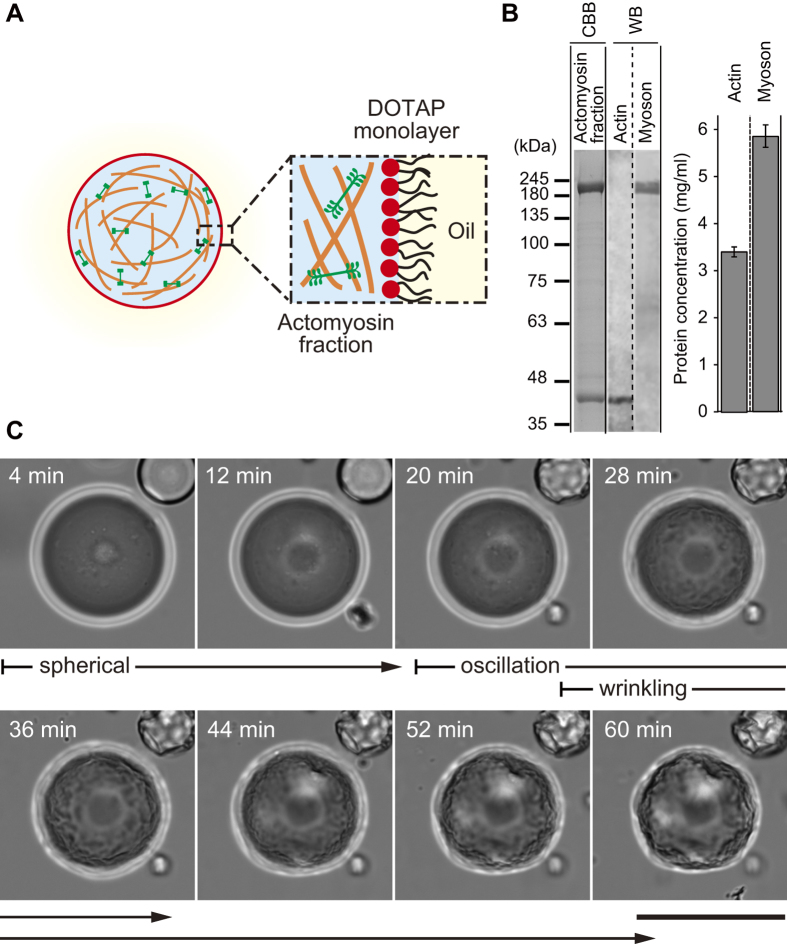
(**A**) Schematic illustration of a cell-sized water droplet of the actomyosin fraction, enclosed with a lipid monolayer. (**B**) Characterization of the actomyosin fraction; error bars represent the standard error of the mean (*n* = 5). (**C**) Dynamic deformation of the lipid interface owing to the contraction of the confined actomyosin fraction. The interface began to oscillate at around 30 min. After the oscillation started, the wrinkle grew on the interface. Scale bar is 50 μm.

Figure 1A shows a schematic illustration of the present model system, a cell-sized water-in-oil droplet encapsulating actin and myosin II extracted from *Amoeba proteus* as shown in Fig. 1B (see Methods for detail). As a non-specific interconnection between the actomyosin and the inner surface of the membrane, we adapted electrostatic attraction between negatively-charged actin together with myosin as an actomyosin complex and positively-charged lipid DOTAP at the oil/water interface. The suitable conditions for the dynamic deformation were examined with respect to the concentrations of actin and myosin, as well as the fraction of DOTAP. The concentration-dependent actin distribution inside a droplet confirmed that 3 mg/ml actin was the most suitable to form the uniform cortex-like structure attracted at the interface by the pure DOTAP monolayer, while the higher concentration of actin than 3 mg/ml led to homogeneous distribution inside the aqueous phase (Fig. S1). Accompanied with 3 mg/ml actin, 6 mg/ml myosin caused the interfacial deformation the most efficiently (Fig. S2). Compared to the result in the case where myosin was absent (Fig. S1), this result means that the driving force of the interfacial deformation was exerted by myosin molecules; the actin polymerization and depolymerization were not significant in the present deformation. Figure 1C shows typical time evolution of the transmitted light images of a droplet interface. It should be noted that the deformation in the images visualized by the transmitted light means the deformation emerged not only on the actomyosin cortex-like structure but also on the lipid monolayer at the interface, because the oil/water interface appears with the highest contrast in the transmitted light images. By varying the lipid fraction of positively-charged DOTAP and electrically-neutral phospholipid DOPC, we also confirmed that the pure DOTAP monolayer resulted in the highest percentage of the deformed droplets (Fig. S3). Therefore, we set the condition as 3 mg/ml for actin, 6 mg/ml for myosin, and the pure DOTAP lipid monolayer in the following observations.

### Dynamic interfacial deformation

**Figure 2:**
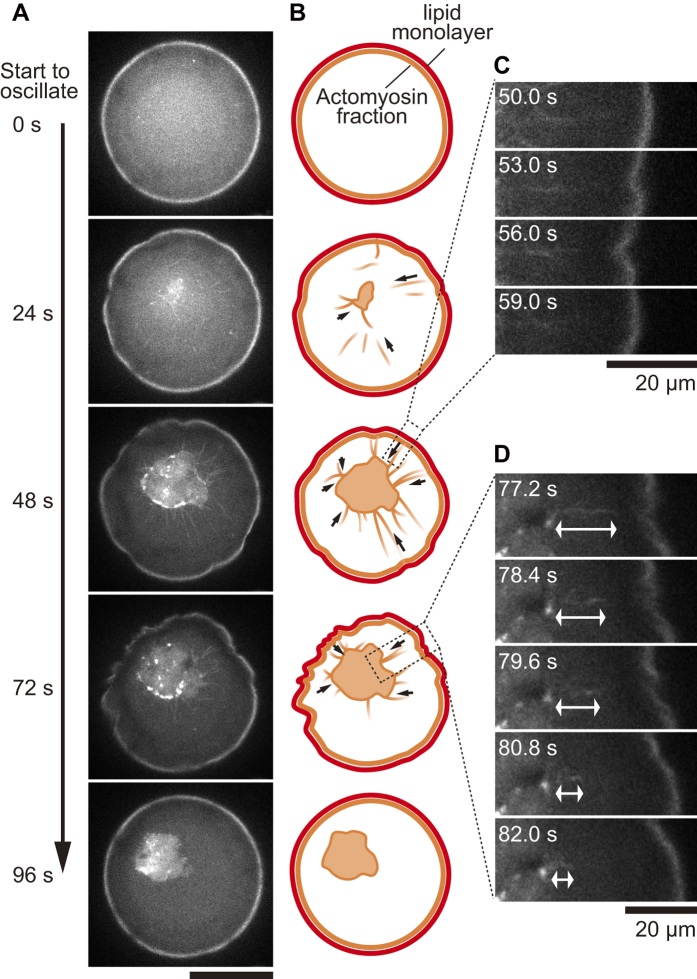
Actin distribution during the non-periodic oscillatory deformation. (**A**) Confocal fluorescence images of the interfacial oscillatory deformation. Actin filaments (fluorescently-labeled) spontaneously form an aster-like structure and pull the lipid interface. Scale bar is 50 μm. (**B**) Schematic illustrations corresponding to the confocal images shown in (**A**) Red and orange colors denote the lipid interface and actomyosin fraction (fluorescently-labeled in **A**), respectively. The arrows indicate the inward direction of bundle motion. (**C**) Magnified images of the region enclosed with the connected dashed line in which an actin bundle pulls and distorts the interface. The rectangular images were rotated to put horizontally. Scale bar is 20 μm. (**D**) Magnified images of a region in which the core pulls an actin bundle toward the center of the droplet, leading to the formation of the aster-like structure. The white arrow indicates the length of the actin bundle in the aqueous bulk. Scale bar is 20 μm.

Next, we investigated the dynamical properties of the obtained interfacial deformation in detail. For a typical case shown in Fig. 1C, in the initial stage, from the preparation to tens of minutes, the droplet remained spherical due to interfacial tension, as is the case with the behavior of general water droplets. Subsequently, the droplet interface exhibited two modes of characteristic deformation. In mode (i), after approximately 20 min from the preparation, several regions of the interface spontaneously started to deform: the interface repeatedly dented with a characteristic size of approximately 10 μm and restored the original spherical shape (Movie S1). This oscillatory deformation continued for several tens of minutes. In mode (ii), after 40 min from the preparation, wrinkles developed on the interface. Although the two deformation modes of (i) the oscillatory behavior and (ii) the wrinkle development tended to occur in this order, the both cases in which the mode (i) and the mode (ii) occurred simultaneously and non-simultaneously were observed in the same sample solution (Figure S4), strongly indicating that these phenomena were independent of each other. Hence, the distinct mechanisms that originate from a contractile property of actomyosin underlie the two different deformation modes. Here, we focus on the dynamic and repetitive interfacial deformation observed as the interfacial oscillation mode (i). To reveal the mechanism that underlies the interfacial oscillation, we confirmed the actin distribution within a droplet using confocal fluorescence microscopy. During the oscillatory deformation, the confocal fluorescence images revealed that initially homogeneous actin filaments within the droplet spontaneously converged into the observable bundles, which further assembled into a three-dimensional aster-like structure in a hierarchical manner (Fig. 2A,B). Based on this microscopic observation, the actin bundles between the interface and the central core were found to cause interfacial deformation by pulling the interface toward the core (Fig. 2C,D, Movie S2). The convergence of the actomyosin bundles connected to the significantly deformed part of the interface could be visualized by chemical fixation as shown in Fig. S5. The results suggest that the mechanical interconnection between the aster structure and the soft interface was mediated by actomyosin network, and the contractile force generated by the mediated actomyosin was converged and transmitted to the lipid interface through the actomyosin bundle as an inward force. Although the formation of an aster-like structure of actomyosin has been previously reported^21^,^22^, the oscillatory deformation of the interface associated with this active behavior accompanied with the structure development is, to the best of our knowledge, reported for the first time. After the oscillations ceased together with the depletion of the actomyosin bundles bridging the aster core and the interface, the tangential tension of the lipid interface leads to regain its original spherical shape. Thereafter, (ii) wrinkle development started on the lipid interface, where the remaining actomyosin fraction exhibited a cortical shell (Movie S2)^44^.

### Symmetry breaking of the interfacial agitation

**Figure 3:**
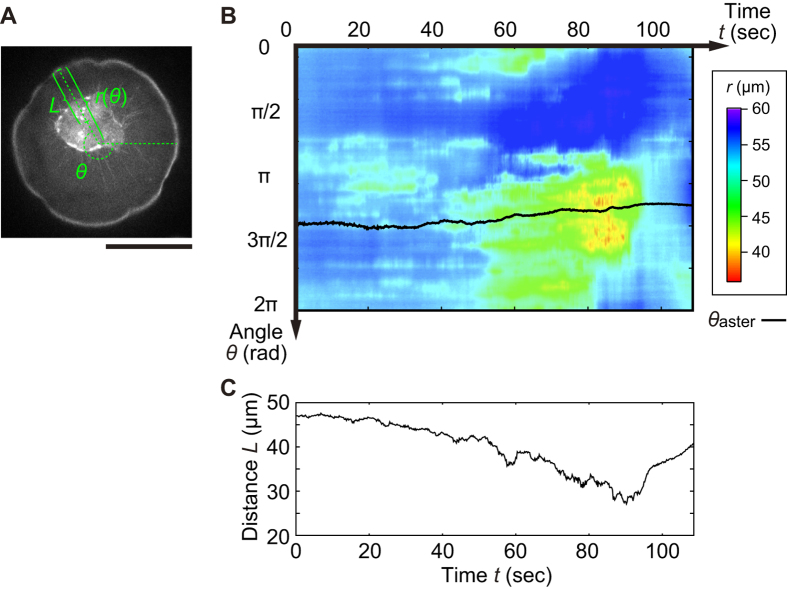
(**A**) Geometrical configuration of the position of the interface and the aster core from the projected droplet center of mass in polar coordinate. Scale bar is 50 μm. (**B**) Color map of the spatiotemporal radial position of the interface 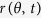. Black line represents the angular position of the aster core 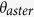. (**C**) Corresponding distance *L* between the radial position of interface 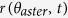 and the center of core as a function of *t*.

It should be noted that the present spherically closed interface provides spontaneous symmetry breaking of the activity of the interface deformation. In the two dimensionally projected polar coordinate (*r*, *θ*) exemplified in Fig. 3A, the spatiotemporal map of the fluctuation amplitude became asymmetric, which was accompanied by the displacement of the aster core toward 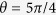 in angular position, whereas in the other angles the fluctuation amplitude remained much smaller (Fig. 3B). Further the distance between the interface and the aster core *L* became close concurrently with and/or subsequently to the displacement of the aster (Fig. 3C). According to the decrease in the distance from the initial state *L*(*t* = 0 s) ~ 47 μm ~ *r*(*t* = 0 s) to *L*(*t* = 90 s) ~ 27 μm, the interfacial agitation in the vicinity of the aster core increased as shown in Fig. 3B. When the aster core reeled in the bundles (*t* = 95 s~) after detaching the actomyosin bundles from the interface, the interfacial fluctuation ceased and the interface was restored to the initial spherical shape. Therefore, the experiments suggest that the shorter *L* results in the stronger attraction with larger fluctuation amplitude. Since the distance between the core and the interface is also defined by the size of the droplet, the deformation behaviors as a function of the size were analyzed, where the deformation amplitude was found to be proportional to the droplet size according to the lower Laplace pressure in larger droplets (see Figure S6 for detail).

### Molecular contributions to the force generation

**Figure 4:**
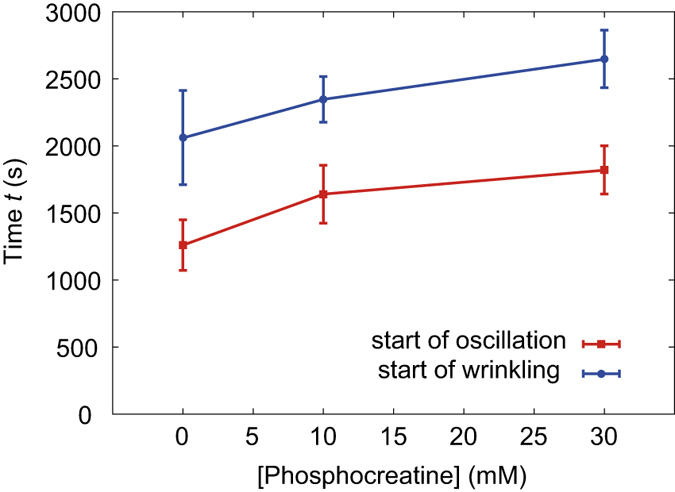
Phosphocreatine-concentration dependence of the onset time of the deformation. Squares (red): the onset time of the non-periodic oscillatory deformation; circles (blue): the onset time of the wrinkle development. Error bars represent the standard error of the mean (*n* = 5 for each phosphocreatine concentration).

It is well known that the contractile force^12^,^13^ and aster structural formation^21^ are induced by motor proteins in the presence of the crosslinkers of actin network. Although our experimental system was mostly composed of actomyosin (Fig. 1B, over 85%), a certain amount of other proteins including actin crosslinker proteins is contained. In addition, highly concentrated myosin bipolar thick filament^26^,^27^ or myosin strongly bound to actin in ATP depleted condition, *i.e.*, rigor state myosin^13^,^22^,^28^,^29^, can also play as effective crosslinkers to generate contractile force in the actin network. Since the myosin concentration in the present study, 6 mg/ml, is much higher than the critical assembling concentration, ~0.5 μM (*e.g.*, 0.11 mg/ml of skeletal muscle myosin), myosin thick filaments emerge in the present condition, which were previously confirmed by electron micrograph^30^. To make clear the contribution of ATP depletion in the present system, we checked the behavior in the presence of ATP regeneration system (0 mM, 10 mM, and 30 mM in phosphocreatine concentration) (Fig. 4). Consequently, we confirmed that the time point when the oscillatory deformation starts was clearly delayed according to higher phosphocreatine concentration. In this concentration region, the same mechanism of the oscillatory deformation, *i.e.*, the aster formation inside the droplet, was observed (Fig. S7). We also confirmed that the second deformation mode, wrinkle development, was also delayed according to the higher phosphocreatine concentration. The time delay of the oscillatory deformation means that the force generation is correlated to the gradual increase of the amount of rigor state myosin, which possibly acts as one of effective crosslinkers.

## Discussion

The time-evolution shown in Movie S2, again, strongly suggests that (i) the oscillation accompanied by the formation of the aster-like structure occurred independently of (ii) the wrinkling deformation, even though the driving force behind both modes was in common, *i.e.*, actomyosin contraction. The independence of the two modes observed here was also confirmed by checking the time for each mode to start for a number of droplets (Figure S4). The result also indicates that start time in each mode and droplet was dispersed especially in smaller droplets. This behavior would correspond to the dispersion of the initial condition, *i.e.*, the encapsulation concentration of the huge macromolecules inside the droplet would be more dispersed in small droplets, whereas that inside the larger droplets converges due to the law of large numbers.

The resultant interfacial deformation indicates that electrostatic attraction is sufficient to maintain the connection between the cortex and the lipid interface, leading to interfacial deformation; furthermore, the result suggests that the force generation and cross-linking between the bundles in the actomyosin core of the aster-like structure are more prominent than those underneath the lipid interface, probably due to the dimensionality of the actomyosin structure, *i.e.*, the aster-like aggregation in the aqueous phase is three-dimensional structure with more cross-links than the cortex-like two-dimensional structure underlying the interface. The more cross-links are formed in the case of the shorter distance between the aster core and interface, which could result in the asymmetric force generation. Regarding the typical energy cost to cause the interfacial deformation, it is remarkable that the energy cost of the deformation in the present interface is 10^3^–10^4^ times higher than that of an elastic lipid bilayer membrane in the case of cell-sized systems^31^,^32^,^33^. By estimating the lower limit of the pulling force in a single indentation that typically exhibits 10 μm in depth from the initial spherical shape, a normal force at least of the order of 10–100 nN is required against the interfacial tension of the order of 1–10 mN/m^32^,^33^. Although not only surface tension but also the volume constraint and elasticity of the cortex structure could contribute to the restoring force, the above simple estimation indicates that at least tens or hundreds of thousands of myosin motors are involved in the generation of force in an actomyosin bundle^34^. Since the aster structure was attracted to the various directions by the contractile forces of the bundles depending on the distance from the interface, the center position should be unstable fixed point leading to the spontaneous symmetry breaking of the shape and the activity of the interfacial agitation. In other words, the contraction of the actomyosin bundle between the aster core and the corresponding interface resulted in three dimensional “tug of war” issue, which determines the central position under the pulling forces toward different directions. Once the pulling force is reduced in one side, the overall force becomes imbalance, leading to further force imbalance.

**Figure 5:**
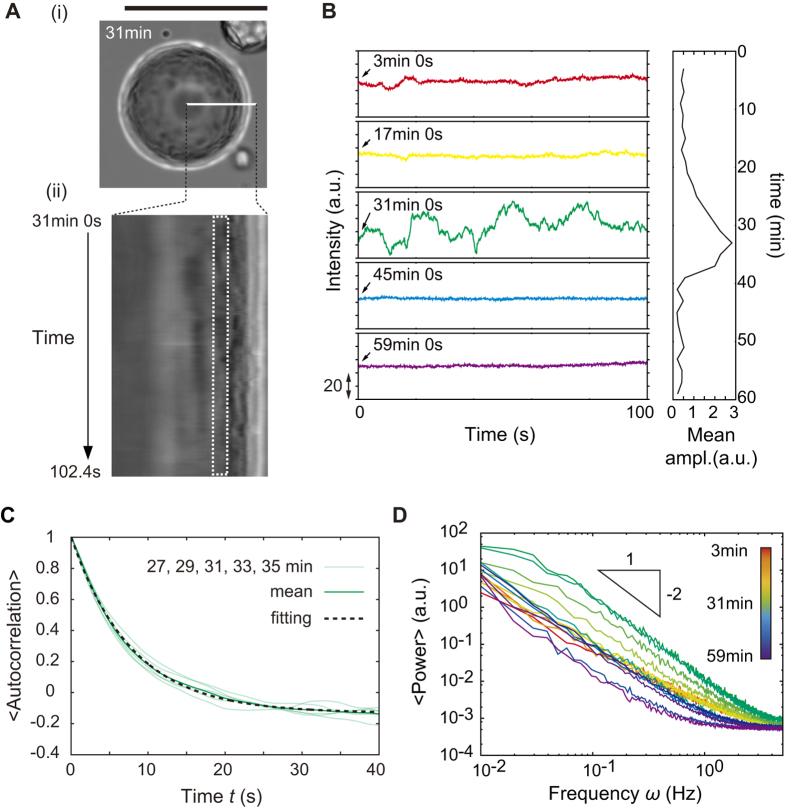
Characterization of the time correlation of the dynamic non-periodic oscillation. (**A**) Example of the time course of the intensity oscillation during the non-periodic oscillatory deformation. (i) Transmitted light images of the droplet at 31 min from the droplet preparation. Scale bar is 50 μm. (ii) Spatiotemporal map of a magnified region indicated in (i) by the radial white line. The white broken box in (ii) corresponds to the region in-focus, which were analysed. (**B**) (left) Time course of the intensity at various time points ranging from 3 min to 59 min. (right) Mean amplitude of the oscillation profiles exemplified in the left graphs. Time-dependent oscillation occurred only around 30 min. (**C**) Autocorrelation of the oscillatory deformation around 30 min (27, 29, 31, 33, 35 min, and the mean of them). The broken line is the result of exponential fitting of the mean. (**D**) Temporal variation in power spectra. There is no peak for the broad frequency domain *ω* = 0.01–5 s^−1^, indicating the temporal oscillatory deformation is non-periodic.

**Figure 6:**
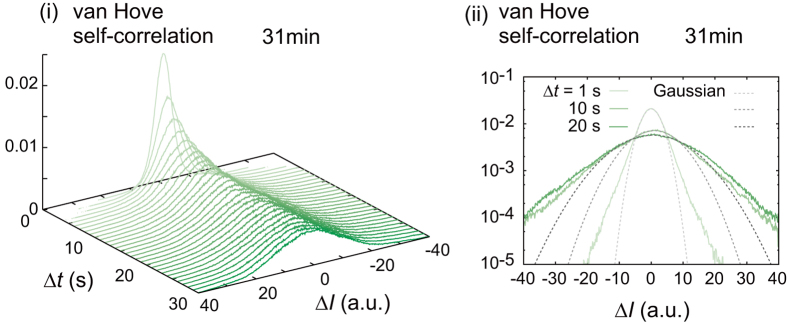
The van Hove self-correlation function obtained for the oscillatory deformation. (ii) The tails of the distributions are almost linear in single logarithmic plots, *i.e.*, exponential distributions. Gray broken lines are Gaussian distributions corresponding to the green lines with the same brightness. All fittings were conducted around the peaks of the experimental distributions.

We here characterized (i) the oscillatory deformation of the interface by analyzing the intensity profiles of the transmitted light images. The intensity of transmitted light reflects the degree of the shape change of the curved surface, whereas confocal fluorescence images (as in Fig. 2) are unsuitable for visualization of the present fast dynamics and long-term tracing due to the impact of fluorescence photo-bleaching. Prior to the following analyses, approximately linear relationship between the transmitted light intensity and the interfacial deviation was confirmed (Fig. S6A). A time-sequential intensity profile along the white line shown in Fig. 5A (i) is presented as a kymograph (Fig. 5A (ii)). The rectangular region clipped by a white dotted line indicates the in-focus region in which we analyzed the time course of intensity. The intensity profile at various time points (3 min, 17 min, 31 min, 45 min and 59 min after the droplet preparation) clearly shows that the oscillatory deformation occurred for 20 min at around 30 min in this droplet (Fig. 5B).We calculated the autocorrelations of the temporal oscillatory deformation for 360 degrees by rotating the radial white line shown in Fig. 5A (i) in increments of 1 degree, and then angularly averaged them. The angularly averaged autocorrelation, which is denoted by 〈autocorrelation〉, and its exponential fitting revealed that the relaxation time for one pulse-like oscillation was 7.83 ± 0.03 s. The excellent fitting with exponential indicates that the oscillatory deformation shown here is non-periodic (Fig. 5C). Thus, we call the present oscillatory deformation mode as non-periodic oscillation. We also obtained the angularly averaged power spectra at each time point from the preparation of the droplet (Fig. 5D). To grasp the nature of the non-periodic oscillation, we focus on the spectrum at around 31 min (denoted by a green line), when the oscillatory behavior was observed for this droplet. There, both the signal-to-noise ratio and the power spectral magnitude were a few orders of magnitude greater than those in other time regions. The scaling exponent of the power spectrum plotted versus frequency in this region was -2 around the characteristic frequencies of *ω* = 1 Hz for a single pulse and *ω* ~ 0.1 Hz for the characteristic time between pulses. Considering the shift to the gentle slope in the low frequency region, the spectrum can be fitted by the Lorentzian as a function of frequency *ω*. To confirm the rarity of the deformation process by the actin bundles, we calculated the probability density distribution of intensity change Δ*I* versus time lag Δ*t* which is called the van Hove self-correlation function (Fig. 6). In general, if the deviation is uniformly and thermally activated, *i.e.*, under equilibrium thermal fluctuation, the van Hove self-correlation function becomes a Gaussian distribution. However, the van Hove self-correlation function shown here is not Gaussian but rather exponential at the tail of the distribution for various lag times Δ*t*, consistent with the rarity of events of extra-large deviations, *i.e.*, a Poisson process.

The above results can be interpreted as follows: The non-periodic oscillatory deformations of the interface are caused by the normal stress exerted by actomyosin bundles, which is a highly random phenomenon identified as a Poisson process dominated by a small number of bundles. The repetitive nature of the deformation is composed of two factors: contraction of the connected actomyosin bundle inward and restoration of the interface outward accompanied by the mechanical detachment of the bundle. Note that the distribution around the mode value is Gaussian, indicating that the generation of the force by the bundles partly detached from the interface, which exhibit small stick-slip type deviations, causes a large number of small random fluctuation of the interface, whereas the complete detachment of the bundle causes a small number of large pulse-like deviations (Movie S2), which yield the exponential decay of the autocorrelation and Lorentzian function of the power spectrum. A similar distribution for rare events of extra-large deviations has been reported in other complex systems, *e.g.*, anomalously diffusive colloids dispersed in micro-structures such as the actin network, or in jamming states, in which the deviations are controlled by a large number of small thermal deviations and a small number of large non-diffusive jumps due to steric structures^14^,^35^,^36^,^37^.

In the present study, we have observed for the first time the oscillatory deformation of the interface driven by the connected contractile actomyosin. The non-periodic oscillatory behavior should be realized by the interaction among the force generation of actomyosin, elastic response of the interface, and the intrinsic properties in the system such as the geometric constraint and the bundle detachment. To further understand the physical nature of this interfacial deformation, we developed an equation of motion of the interface based on the experimental results. The exponentially decaying autocorrelation and Lorentzian-type power spectrum, which are the statistical nature of the non-periodic oscillatory deformation of the interface, are generally derived from over-damped Langevin equation of a mass-less area element:


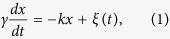


where the left hand side is damping factor proportional to the interfacial velocity with damping coefficient *γ*, the first term in right hand side is restoring force by effective spring with spring constant *k*, and *ξ*(*t*) is external random force, which satisfies the mean 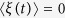 and the autocorrelation function 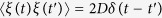 (*D*; amplitude of the external random force)^38^. The Gaussian peak and the exponential decay in the tale of van Hove self-correlation function (Fig. 6) clearly indicate that the distribution of 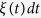 is composed of not only Gaussian but also Poisson distribution^39^,^45^. The corresponding random force is the linear combination of white Gaussian noise 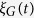 (mean; 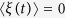, variance; 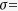
const.) and white Poisson noise 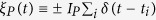 (noise intensity; 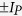  = const., birth rate; *λ* = const.), which satisfy 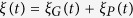 and still keep the property of white noise as 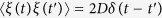, where 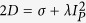 = const. In the present non-periodic oscillation, these restoring and damping forces are balanced with the driving force from active actomyosin contraction, which is here assumed as random force. By solving the above differential equation, we reproduce the exponential decay of the spatial autocorrelation equation


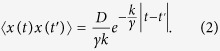


By fitting the experimentally obtained autocorrelation function with this theoretical autocorrelation equation, we obtain the ratio between the spring constant and the damping coefficient as *k*/*γ* ~ 0.1 s^−1^, which coincides with the inverse of characteristic relaxation time. From a physical viewpoint, the damping term would correspond to viscous friction against the active interfacial velocity. On the other hand, the linear spring term would correspond to the linear-responsive confinement for sufficiently small elastic deviation of an area element from adjacent interfaces. Although the viscous damping should be observed also in the previously reported bulk systems^13^,^14^,^15^, it is remarkable that the emergence of the elastic restoring force is an essential nature of the closed spherical interface, resulting in a spatial constraint for the deviation. Therefore, we concluded that the present non-periodic but oscillatory deformation of the interface is realized by the combination of the closed spherical geometry and the Poisson events of the stochastic contraction and detachments of actomyosin.

We have reported the dynamic and continual interfacial deformation caused by actomyosin contractility. The initially homogeneous actomyosin solution encapsulated in a cell-sized water-in-oil droplet spontaneously causes the two distinctive deformation of the droplet interface, *i.e.*, non-periodic oscillation and interfacial wrinkling. These phenomena are strongly coupled with structural formation of the actomyosin, aster-like and cortex-like structures, respectively, reflecting the active self-assembly under cell-sized confinement. In our experimental system, it is remarkable that the non-periodic oscillation of the interface is realized by the restoring force due to the closed confinement, as well as the percolation of the stochastic contractile force, *i.e.*, the bridge formation, across the lipid interface and the central actomyosin structure as a Poisson process. As a future work, the elucidation of the detailed molecular mechanisms of the force generation, e.g., crosslinking, actin binding proteins, motor duty ratio, etc. in the present system would be important. Particularly, how much amount of each candidate of crosslinkers, such as myosin biopolar thick filaments, rigor state myosin, and actin crosslinker proteins, contributes to the actual level of crosslinking would be essential information. It should be noted that the present system contained up to 15% unknown proteins, which probably included unspecified actin crosslinker proteins. To determine the amount of the existing actin crosslinker proteins in the present system should lead to better understanding of microscopic contributions to the macroscopic/mesoscopic structure formation and interfacial deformation caused by the emergent contractile force. The resultant dynamic deformation of the interface provides the experimental evidence that both the magnitude of force generation and the structural formation of the components involved in the force generation are crucially important to determine what type of spatiotemporal dynamics appears in a deformable system with force generation and force transduction. Our present findings demonstrate that the percolation of the active forces throughout the cell-sized three-dimensional space by the three-dimensional structural formation of actomyosin can drive the stochastic oscillatory deformation of the interface.

## Methods

### Cell Culture

According to previous report^40^, *Amoeba proteus* was cultured in KCM medium (7 mg/l KCl, 8 mg/l CaCl_2_, and 8 mg/l MgSO_4_-7H_2_O) at 25 °C and fed with *Tetrahymena pyriformis* twice a week. To avoid contamination of undigested *T. pyriformis* cells were starved for at least 3 days before use.

### Preparation of the actomyosin fraction

All the following preparations were performed at 2 °C to avoid disfunction of actin and myosin proteins. The actomyosin fraction was prepared by modifying a previously described method^30^. A portion of *A. proteus* (10 g) suspended in a cell washing solution (2 mM *O*,*O′*-Bis(2-aminoethyl)ethyleneglycol-*N*,*N*,*N′*,*N′*-tetraacetic acid (EGTA), 2 mM MgCl_2_, and 20 mM Piperazine-1,4-bis(2-ethanesulphonic acid) (PIPES)-KOH, pH 7.0) was first centrifuged at 6,000 ×_ _*g* for 2 min. After the supernatant was removed, the resultant precipitate was centrifuged at 600,000 × *g* for 20 min to obtain an actin rich solution. Next, the precipitate was suspended in a 3 M KCl solution (3 M KCl, 2 mM MgCl_2_, 1 mM threo-1,4,-dimercapto-2,3-butanediol (DTT), 20 mg/ml leupeptin, 20 mg/ml pepstatin A, 0.2 mM phenylmethylsulphonyl fluoride (PMSF), 20 mM imidazole-HCl, pH 7.0) and centrifuged at 400,000 ×_ _*g* for 10 min. The obtained supernatant was dialyzed against 50 mM KCl, 2 mM EGTA, 2 mM MgCl_2_, 1 mM DTT, 0.2 mM PMSF, 20 mM imidazole-HCl, pH 7.0 for 5 h. The dialyzed specimen was centrifuged at 20,000 ×_ _*g* for 5 min, and the precipitate was suspended in a 270 μl EMPA solution (5 mM EGTA, 6 mM MgCl_2_, 1 mM DTT, 2 mM adenosine-5*′*-triphosphate (ATP), 30 mM PIPES-KOH, pH 7.0), and the actin rich solution (30 μl) was added. The resultant fraction dissolving the actomyosin contains 4.5 mM EGTA, 5.4 mM MgCl_2_, 0.9 mM DTT, 1.8 mM ATP and 27 mM PIPES. The protein concentration in the actomyosin fraction was determined by Bradford assay with bovine serum albumin as the standard, where the measured concentration of actin and myosin were 3 mg/ml and 6 mg/ml respectively. In addition, we also confirmed the concentrations of these proteins using absorbance at 280 nm and muscle actin/myosin extinction coefficients. The estimated values of actin and myosin concentration were 2.3 mg/ml and 6.2 mg/ml, which corresponded to 2.55 and 2.73 of absorbance of actin and myosin, respectively. The concentration of actin and myosin were calculated from total protein concentration and ratio of actin or myosin to total proteins using SDS-PAGE and the Fiji software package (http://fiji.sc/wiki/index.php/Fiji). Actin and myosin were determined by immunoblotting using anti-β-Actin (Poly6221; BioLegend) and anti-Myosin II from *A. proteus*. Horseradish peroxidase (HRP)-conjugated secondary antibodies (Poly4053; BioLegend and Poly4064; BioLegend). In the experiments with ATP regeneration system, 0.2 mg/ml creatine phosphokinase (Sigma aldrich) and 0 mM to 30 mM phosphocreatine (Nacalai tesque) were added as final concentration in the actomyosin fraction.

### Preparation and observation of the actomyosin droplets

Cell-sized water-in-oil (W/O) droplets with a lipid monolayer were prepared using a previously described method^41^,^42^. In brief, 1,2-dioleoyl-3-trimethylammonium-propane chloride (DOTAP, Avanti Polar Lipids) was dissolved in chloroform at a concentration of 10 mM. The chloroform was dried at a bottom of a glass tube under a nitrogen stream and put under vacuum overnight to form a dry film of lipids. Mineral oil (Nacalai tesque) was added to the films and then sonicated at 60 °C for 60 min, resulting in dispersed 1 mM DOTAP in oil. Finally, 3% (vol./vol.) actomyosin fraction was added to the oil solution with lipids, and emulsification was performed by vortexing and pipetting to form cell-sized W/O droplets of actomyosin fraction. The obtained droplets were observed using an optical microscope (Eclipse Ti, Nikon) at room temperature and recorded using a sCMOS camera (ORCA-Flash4.0, Hamamatsu). The distribution of actomyosin fraction inside a droplets was observed by the actin filaments stained with 10 nM Acti-stain 488 phalloidin (Cytoskeleton). Note that the phalloidin prevents actin filaments from depolymerization. Since the presence and absence of phalloidin did not influence on the oscillatory and wrinkling behaviors, actin polymerization and depolymerization were not crucial in the present repetitive deformations. Time-lapse images of the stained actin filaments were collected using a microscope (IX71, Olympus) equipped with an EM-CCD camera (iXon, Andor) and a confocal scanner unit (CSU-X1, Yokogawa). In the experiments of droplets in the absence of myosin molecules (Fig. S1), a mixture of 300 μl EMPA solution and 30 μl actin rich solution was used in place of the actomyosin fraction in the above-described procedure. To visualize the actomyosin structure near the interface during the non-periodic oscillation regime, actomyosin droplets stained by fluoresceinated lifeact peptide^43^ and by fluorescent lipids were fixed by formalin at the oscillatory period to be observed by confocal fluorescence microscope. In detail, equal volumes of formalin (15%(w/w)) and mineral oil were well mixed and centrifuged at 6,000 × *g* for 5 min. Supernatant was collected and preserved at 2 °C, which was named a fixing oil. The oscillating actomyosin droplets, which stained by 1 μg/ml Carboxytetramethylrhodamine-lifeact (purchased from Eurofinsgenomics) and 2.5 μM ATTO 488 labeled DOPE (ATTO-TEC), were prepared by above mentioned method and cooled on ice after checking the occurrence of oscillation. Equal volume of fixing oil to the specimen was gently added, mixed and left for 30 min on ice. Then fluorescence of actin and interface were observed with a microscope (IX71, Olympus) equipped with a sCMOS camera (Zyla 4.2, Andor) and a confocal scanner unit (CSU-X1, Yokogawa) at room temperature.

## Additional Information

**How to cite this article**: Nishigami, Y. *et al.* Non-periodic oscillatory deformation of an actomyosin microdroplet encapsulated within a lipid interface. *Sci. Rep.* **6**, 18964; doi: 10.1038/srep18964 (2016).

## Supplementary Material

Supplementary Information

Supplementary Movie S1

Supplementary Movie S2
