# Supplementary material for: Non-periodic oscillatory deformation of an actomyosin microdroplet encapsulated within a lipid interface
Source: Sci Rep. 2016 Jan 12;6:18964. doi: 10.1038/srep18964 (PMC4709586; doi:10.1038/srep18964)
Supplement: Supplementary Information [file srep18964-s1.pdf]

Non-periodic oscillatory deformation of an actomyosin microdroplet encapsulated within a lipid interface

Yukinori Nishigami[1,+], Hiroaki Ito[1,+,*], Seiji Sonobe[2], and Masatoshi Ichikawa[1,*]
[1]Department of Physics, Graduate School of Science, Kyoto University, Kyoto 606-8502, Japan
[2]Department of Life Science, Graduate School of Life Science, University of Hyogo, Harima Science Park City, Hyogo 678-1297, Japan

*ito@chem.scphys.kyoto-u.ac.jp (H.I.); ichi@scphys.kyoto-u.ac.jp (M.I.)
[+]these authors contributed equally to this work

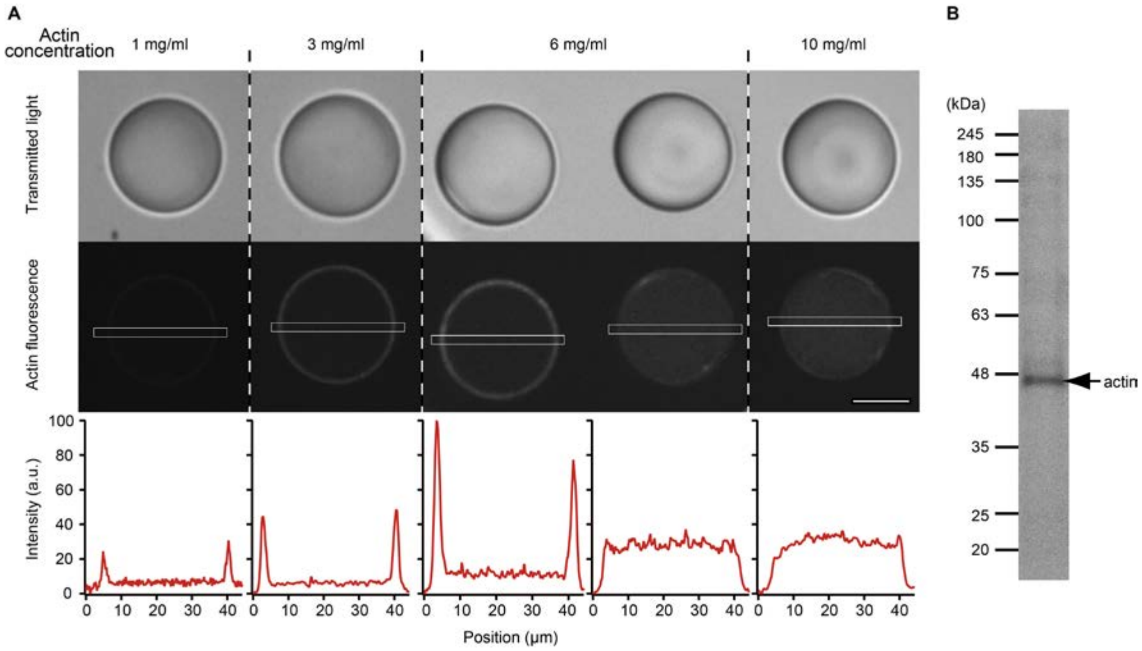

Figure. S1. Various actin distributions inside the droplets at actin concentrations 1 mg/ml, 3 mg/ml, 6 mg/ml and 10 mg/ml. (Top) Transmitted light images, (Middle) the corresponding confocal fluorescence images of actin distributions, and (Bottom) fluorescence intensity distribution in the regions enclosed with the white boxes on the above confocal fluorescence images. 3 mg/ml actin effectively formed actin cortex underlying the interface, while at the higher concentrations actin tends to be homogeneous inside the droplets. Note that the droplets did not deform because myosin was absent. Scale bar is 20 μm.

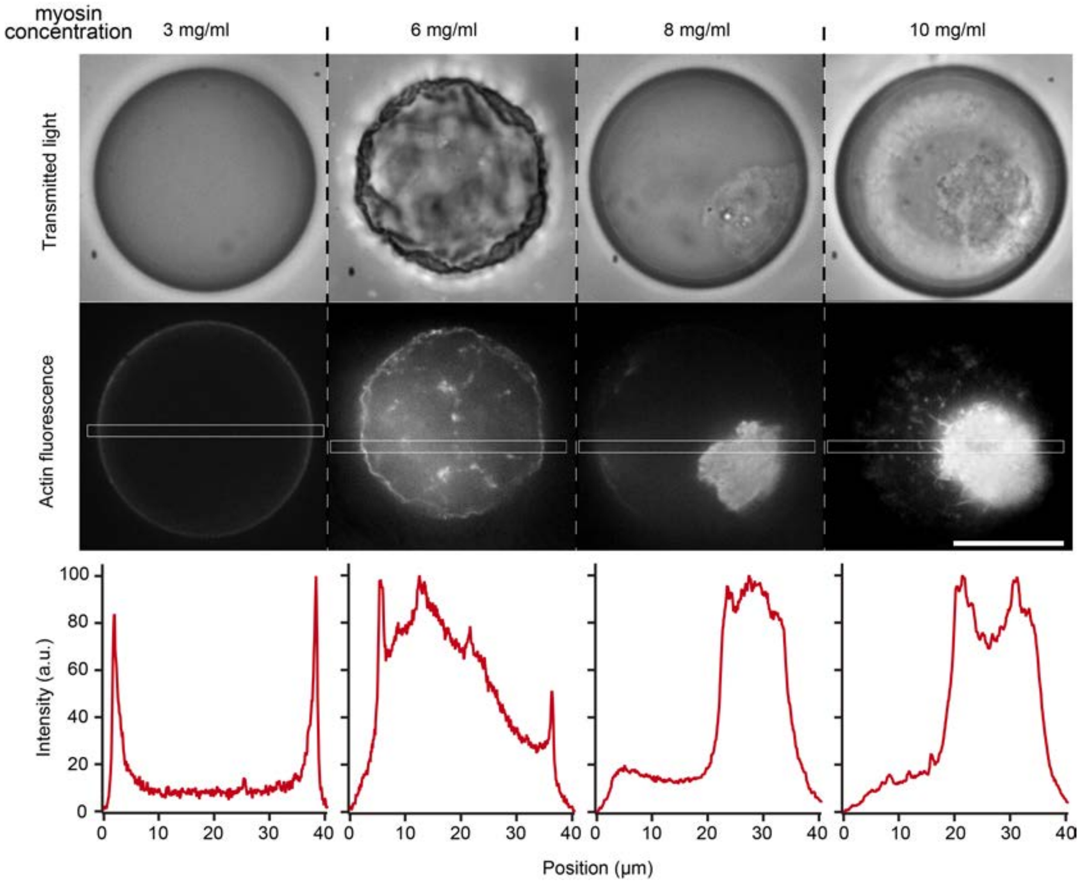

Figure. S2. Various actin distributions in the presence of myosin at myosin concentrations 3 mg/ml, 6 mg/ml, 8 mg/ml and 10 mg/ml. (Top) Transmitted light images, (Middle) the corresponding confocal fluorescence images of actin distributions and (Bottom) fluorescence intensity distribution at the regions enclosed with the white boxes on the above confocal fluorescence images. Actin concentration is fixed as 3 mg/ml. 6 mg/ml myosin effectively caused contraction and interfacial deformation, while it did not or it detached and aggregated at the aqueous phase in the cases of lower or higher myosin concentrations, respectively. Scale bar is 20 μm.

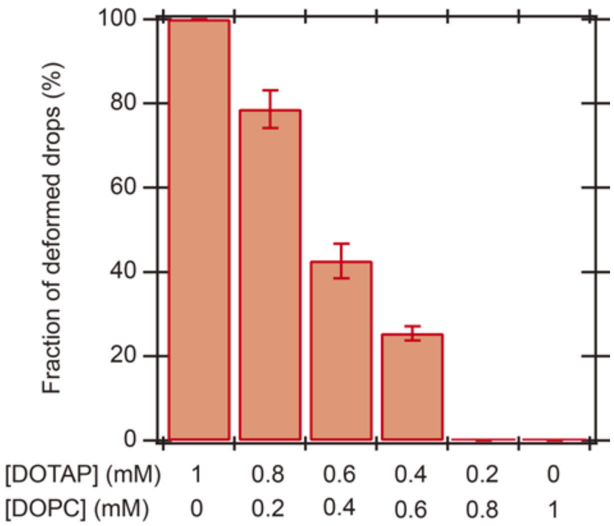

Figure. S3. Fraction of deformed droplets at the lipid concentrations DOTAP : DOPC = 1 mM : 0 mM, 0.8 mM : 0.2 mM, 0.6 mM : 0.4 mM, 0.4 mM : 0.6 mM, 0.2 mM : 0.8 mM and 0 mM : 1 mM. The total lipid concentration was fixed as 1 mM, Decrease in DOTAP concentration resulted in decrease in the deformation fraction. Error bar represents standard error of the mean calculated from 5 experiments.

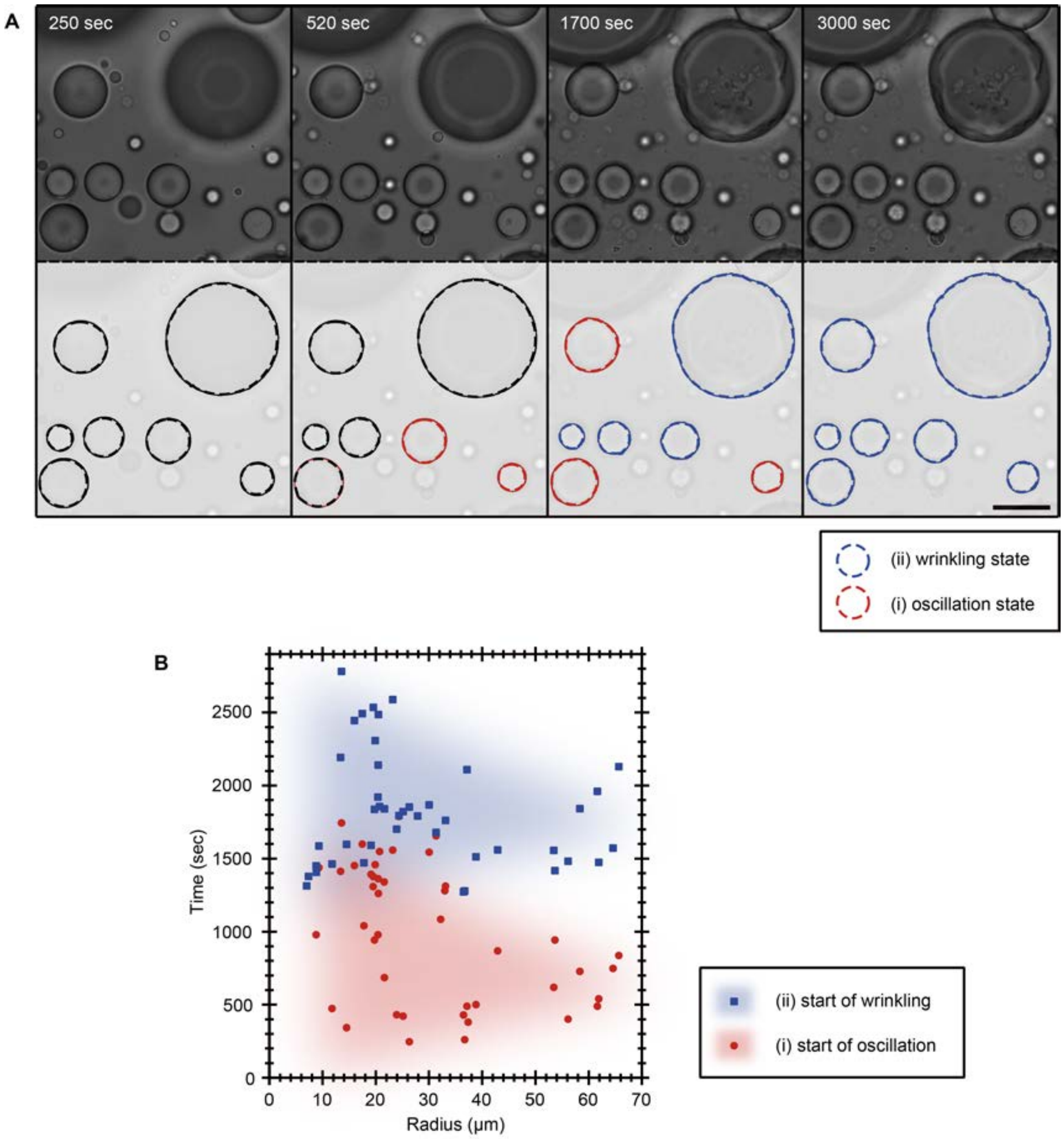

Figure. S4. (A) Wide view of the transmitted light observation. All the droplets deformed under the appropriate condition: [actin] = 3 mg/ml, [myosin] = 6 mg/ml, and 1 mM pure DOTAP monolayer. Scale bar is 50 μm. (B) Beginning times of the two deformation modes (i) oscillation and (ii) wrinkling for various sized droplets.

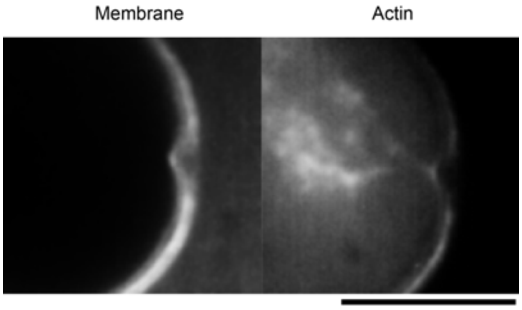

Figure. S5. Connective behavior between the actomyosin bundles and the cortex underneath the surface of the droplet. The droplets were chemically fixed at the time region of the oscillation and then observed by confocal microscopy. The actomyosin bundles were contacted onto the actomyosin cortex at the deformed part of the droplet. Scale bar is 10 μm.

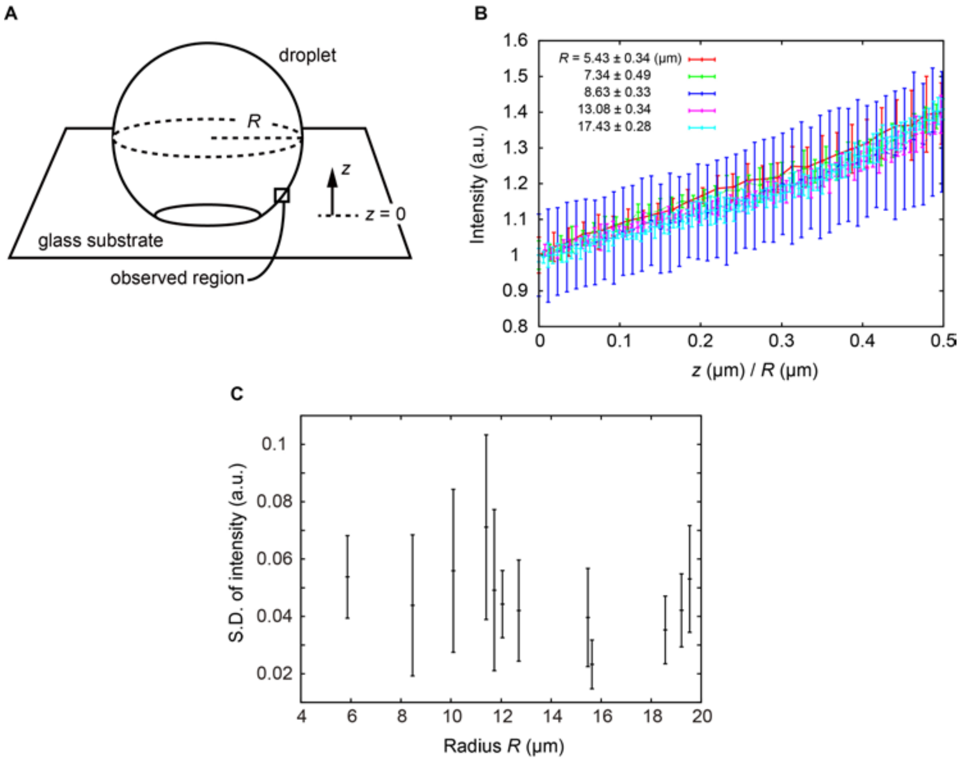

Figure. S6. (A) Schematic illustration of the calibration measurement for the intensity-deviation relationship. (B) Calibration of the relationship between the normalized intensity of brightness I and the displacement z normalized by each droplet radius R. The range $z/R = 0 - 0.5$ corresponds to the observed lower hemisphere. The values and error bars represent mean ± standard deviation (S.D.) coincide with the measurement of three droplets for each radius. The linear intensity-displacement relationship is universal for various droplet sizes. The slope of the master curve is 0.8. (C) S.D. of the normalized oscillatory intensity as a function of droplet radius R. The normalized deformation amplitude is independent of the droplet radius R, *i.e.*, the absolute amplitude is roughly proportional to the radius R. Based on the calibration in (B), the mean normalized amplitude ≈ 0.05 (a.u.) corresponds to the absolute amplitude ≈ $0.0625R$ (μm), which agrees well with that seen in Fig. 2 (~ 5 μm amplitude and 50 μm radius).

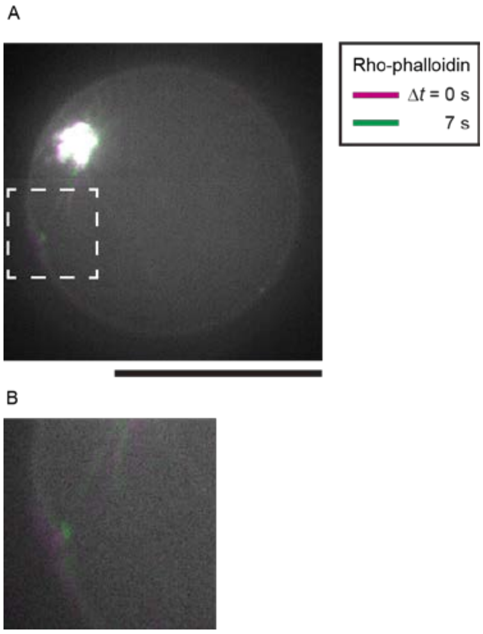

Figure S7. Confocal fluorescence image of the oscillatory deformation in the presence of 30 mM phosphocreatine as ATP regenerating system. (A) Overlay of two images taken after Δt = 7 s. Magenta: Δt = 0 s; Green: Δt = 7 s. Two images are processed by rolling average method over ± 3 s, corresponding to the average over 7 images. Scale bar is 50 µm. (B) Magnified image of a region in which the interfacial deformation is induced by the pulling force by actomyosin bundle, shown in the white dashed box in (A). In the presence of ATP regenerating system, the interfacial deformation is caused by the same mechanism, aster-like structure formation of actomyosin.

Supplementary Movie Legends

Movie S1. Dynamic interfacial deformation throughout the complete process, corresponding to Figure 1C in the main text. There are two distinct deformation regimes: non-periodic oscillation and wrinkle development. Movie is at 4×real time. Scale bar is 50 μm.

Movie S2. Time evolution of actin distribution during the non-periodic oscillation, corresponding to Figure 3A in the main text. The movie was captured by confocal fluorescence microscopy. Movie is at real time. Scale bar is 50 μm.
